# The Effect of Obesity on the Availabilities of Dopamine and Serotonin Transporters

**DOI:** 10.1038/s41598-018-22814-8

**Published:** 2018-03-21

**Authors:** Su Bong Nam, Keunyoung Kim, Bum Soo Kim, Hyung-Jun Im, Seung Hun Lee, Seong-Jang Kim, In Joo Kim, Kyoungjune Pak

**Affiliations:** 1 Department of Plastic Surgery, Pusan National University Yangsan Hospital, Yangsan, Republic of Korea; 2 Department of Nuclear Medicine and Biomedical Research Institute, Pusan National University Hospital, Busan, Republic of Korea; 3 Department of Nuclear Medicine and Research Institute for Convergence of Biomedical Science and Technology, Pusan National University Yangsan Hospital, Yangsan, Republic of Korea; 4 Graduate School of Convergence Science and Technology, Seoul National University, Seoul, Republic of Korea; 5 Department of Family Medicine, Pusan National University Hospital, Busan, Republic of Korea

## Abstract

The authors investigated relations between obesity, age, and sex and the availabilities of striatal dopamine transporter (DAT) and extrastriatal serotonin transporter (SERT) by ^123^I-FP-CIT single-photon emission computed tomography. The study population consisted of 192 healthy controls with screening ^123^I-FP-CIT scans. Specific bindings of ^123^I-FP-CIT to DAT and SERT were calculated using regions of interest. Specific binding ratios (SBRs) of DAT and SERT except pons (r = 0.2217, p = 0.0026), were not correlated with body mass index (BMI). SBRs of midbrains correlated negatively with the BMIs of obese subjects (r = −0.3126, p = 0.0496), and positively with the those of non-obese subjects (r = 0.2327, p = 0.0053). SBRs of caudate nucleus (r = −0.3175, p < 0.0001), striatum (r = −0.226, p = 0.0022), and thalamus (r = −0.1978, p = 0.0074) reduced with age, and SERT availability was higher in males. However, DAT availability was similar in males and females. In conclusion, obesity has an effect on midbrain SERT availability. In addition, BMI was correlated with pontine SERT availability but not with striatal DAT availability. SERT availability was higher in males, but DAT availability showed no gender predilection.

## Introduction

Over half of adults are overweight and 19.5% of the adult population are obese in Organisation for Economic Co-operation and Development member countries^1^. Furthermore, obesity is a risk factor for several malignancies including colon^2^, pancreatic^3^, thyroid^4^, hepatic^5^, and uterine^6^ cancer and for cardiovascular diseases^7^, and diabetes mellitus^8^. Obesity is due to a loss of the balance between energy intake and expenditure over long periods of time^9^, and the brain plays a critical role in controlling and inhibiting the pre-potent responses to foods^9,10^.

Dopamine and serotonin are neurotransmitters involved in the regulation of food intake and body weight^11,12^. Previous studies have used ^123^I-FP-CIT to investigate the role of dopamine transporter (DAT) in striatum^13^ and of serotonin transporter (SERT) in midbrain^14^, pons^15^, thalamus^15^, and hypothalamus^16^. ^123^I-FP-CIT shows high affinity for DAT, and slightly less affinity for SERT^17^. However, as DAT and SERT display nonoverlapping distributions in subcortical structures^14^, ^123^I-FP-CIT enables the co-evaluations of DAT and SERT distributions in a single scan^15^.

The aim of this study was to explore the relations between obesity, age, and sex and the availabilities of striatal DAT and extrastriatal SERT as determined by ^123^I-FP-CIT single-photon emission computed tomography (SPECT) using data obtained from the Parkinson’s Progression Markers Initiative (PPMI).

## Material and Methods

### Subjects

Data used for this article were obtained from the PPMI database (www.ppmi-info.org/data). For up-to-date information on the study, visit www.ppmi-info.org18. The study population consisted of 192 healthy controls that underwent screening ^123^I-FP-CIT SPECT. According to PPMI criteria, males or females aged ≥ 30 years at screening were included. The exclusion criteria applied were; neurological disorder, first degree relative with idiopathic Parkinson’s disease, Montreal Cognitive Assessment score of ≤ 26, medication that might interfere with DAT SPECT scans, anticoagulants that might preclude safe completion of lumbar puncture, investigational drugs, and a condition that precluded the safe performance of routine lumbar puncture. Medical histories, results of neurological examinations (motor and non-motor assessments), and ^123^I-FP-CIT SPECT scans were downloaded.

### ^123^I-FP-CIT SPECT

^123^I-FP-CIT SPECT was performed during screening visits. SPECT scans were acquired 4 ± 0.5 hrs after injecting 111–185 MBq of ^123^I-FP-CIT. Subjects were pretreated with iodine solution or perchlorate prior to injection to block thyroid uptake. Raw data were acquired into a 128 × 128 matrix stepping each 3 or 4 degrees for total projections. Raw projection data were reconstructed using the iterative ordered subset expectation maximization and HERMES (Hermes Medical Solutions, Stockholm, Sweden). Reconstructed images were transferred to pmod (PMOD Technologies LLC, Zürich, Switzerland) for subsequent processing, including attenuation correction.

### Image analysis

Downloaded scans were loaded using pmod v3.6 (PMOD Technologies LLC, Zürich, Switzerland) using a single subject MRI template in Montreal Neurological Institute space^19^. Specific bindings of ^123^I-FP-CIT to DAT and SERT were calculated by region of interest (ROI) analysis. A standard set of volumes of interest (VOIs) defining putamen, caudate nucleus, striatum (putamen + caudate nucleus), and thalamus as described by the Automated Anatomical Labeling (AAL) atlas^20^, and spherical VOIs for pons and midbrain were defined. The cerebellum was chosen as a reference region. A VOI template was applied to measure specific binding ratios (SBRs) of caudate nucleus, putamen, striatum, thalamus, pons, and midbrain as follows; SBR = (target– cerebellum)/cerebellum.

### Statistical analysis

Pearson correlation was used to measure the linear dependences between SBRs and body mass indices (BMIs). For comparisons between obese and non-obese subjects, analyses of covariance was performed using SBR as a dependent variable, BMI as an independent variable, and age as a covariate. The T-test was used to compare the SBRs of males and females. The analysis was performed using GraphPad Prism 7 for Mac OS X (GraphPimad Software Inc, San Diego, CA, USA).

### Data availability

Data used in the preparation of this article were obtained from PPMI database (www.ppmi-info.org/data).

## Results

182 healthy subjects with ^123^I-FP-CIT SPECT from PPMI data were included in this study.

### SBR with BMI

**Figure 1:**
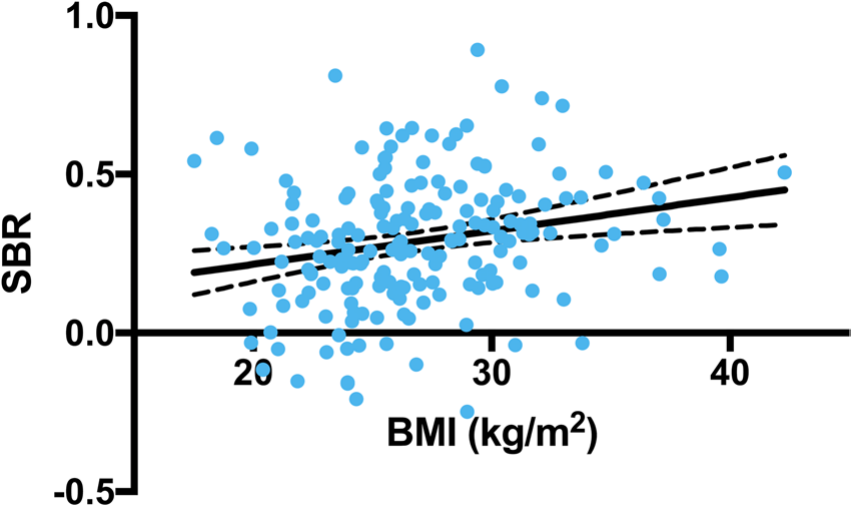
Correlation between SERT availabilities in pons and BMIs (r = 0.2217, p = 0.0026).

**Figure 2:**
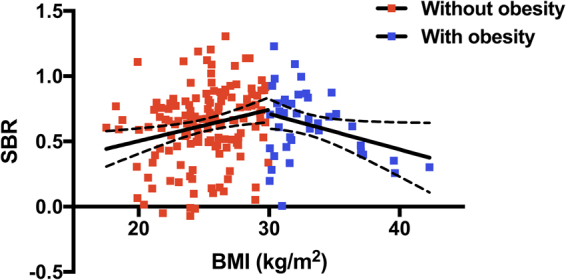
Correlation between SERT availabilities in midbrain and the BMIs of non-obese (r = 0.2327, p = 0.0053) and obese subjects (r = −0.3126, p = 0.0496).

DAT and SERT SBRs were not correlated with subject BMIs, except in pons (r = 0.2217, p = 0.0026, Fig. 1). Subjects were divided into two groups using a BMI cut-off of 30 kg/m^2^: an obese (n = 40) or a non-obese (n = 142) (Table 1). SBRs of midbrain correlated negatively with BMIs of obese subjects (r = −0.3126, p = 0.0496), and positively with the BMIs of non-obese subjects (r = 0.2327, p = 0.0053), and these slopes were significantly different (F = 9.204, p = 0.0028) (Fig. 2). SBRs of pons showed a positive correlation with BMI (r = 0.1968, p = 0.0189) in non-obese subjects. (Table 2). When comparing obese and non-obese subjects, SBR of pons showed significant difference (p = 0.025) with an effect of sex (p = 0.010) and without an age effect (p = 0.153). However, no group difference was found in SBRs of caudate nucleus (p = 0.296), putamen (p = 0.305), striatum (p = 0.293), midbrain (p = 0.847), and thalamus (p = 0.920).

**Table 1:** Subjects’ characteristics.

	Obese (n = 40)	Non-obese (n = 142)	p value
Sex (Male/Female)	27/13	91/51	0.8515
Age	58.2 ± 10.1	61.9 ± 11.4	0.0686
BMI (kg/m^2^)	32.9 ± 3.0	25.1 ± 2.8	<0.0001
Benton test	25.9 ± 5.1	26.2 ± 3.7	0.7123
MOANS score	12.2 ± 3.3	12.4 ± 2.7	0.7661
Epworth Sleepiness Scale	4.9 ± 2.9	5.8 ± 3.5	0.1544
Geriatric Depression Scale	5.0 ± 1.4	5.3 ± 1.4	0.2132
Derived-Letter Number Sequencing	11.4 ± 2.3	11.9 ± 2.9	0.2818
Shared decision making score	47.2 ± 10.4	46.9 ± 10.5	0.8536
UPSIT score	34.1 ± 5.3	33.9 ± 4.9	0.8435

^*^BMI, body mass index; MOANS, Mayo’s Older Americans Normative Studies; UPSIT, University of Pennsylvania Smell Identification Test.

**Table 2:** Correlations between Specific binding ratios and Body mass indices.

SBR	Obese			Non-obese			Total		
	r	95% CI	p value	r	95% CI	p value	r	95% CI	p value
**Dopamine transporter**									
Caudate nucleus	0.0154	−0.2976~0.3253	0.9251	0.0301	−0.1353~0.1939	0.7220	0.1079	−0.0382~0.2494	0.1472
Putamen	−0.06	−0.3647~0.2563	0.7131	0.0643	−0.1015~0.2266	0.4472	0.1008	−0.0453~0.2427	0.1755
Striatum	−0.0231	−0.3322~0.2905	0.8877	0.0490	−0.1167~0.212	0.5625	0.1061	−0.0400~0.2477	0.1540
**Serotonin transporter**									
Midbrain	−0.3126	−0.5687~−0.0012	0.0496	0.2327	0.0707~0.3827	0.0053	0.0828	−0.0634~0.2255	0.2655
Pons	0.0176	−0.2955~0.3273	0.9140	0.1968	0.0332~0.3502	0.0189	0.2217	0.0788~0.3557	0.0026
Thalamus	−0.1381	−0.431~0.1812	0.3955	0.0666	−0.0992~0.2288	0.4309	0.0343	−0.1117~0.1789	0.6454

^*^SBR, Specific binding ratio; CI, confidence interval.

### SBR with Age, and Sex

**Figure 3:**
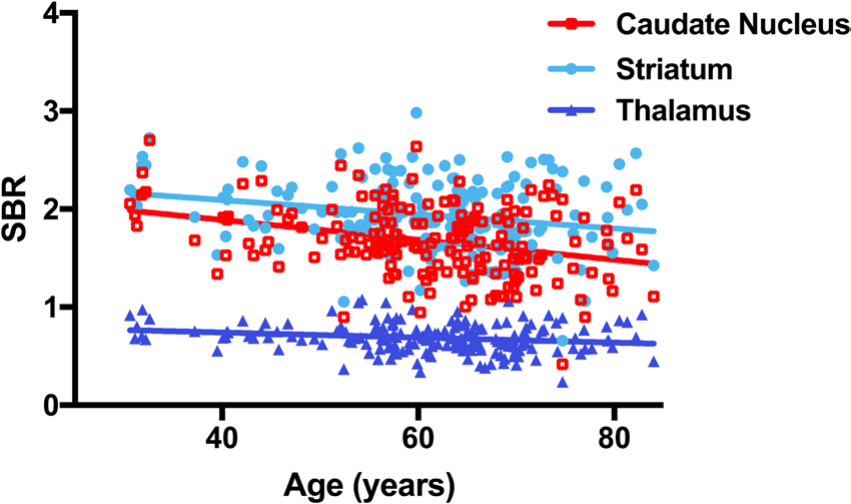
Correlations between DAT availabilities in caudate nucleus (r = −0.3175, p < 0.0001), striatum (r = −0.226, p = 0.0022) and between SERT availabilities in thalamus (r = −0.1978, p = 0.0074) and age.

SBRs of caudate nucleus (r = −0.3175, p < 0.0001), striatum (r = −0.226, p = 0.0022), and thalamus (r = −0.1978, p = 0.0074) showed a reduction with aging (Fig. 3). When the 182 study subjects were divided according to sex, SBRs of DAT in caudate nucleus (p = 0.3949), putamen (p = 0.2403), and striatum (p = 0.2987) were no different, but SBRs of SERT in midbrain (p < 0.0001), pons (p = 0.0068), and thalamus (p = 0.0113) were significantly higher in men than in women.

## Discussion

To the best of our knowledge, this is the largest study undertaken to investigate the effect of obesity on the availabilities of DAT and SERT in healthy controls. In this study, obesity has an effect on midbrain SERT availability. Striatal DAT availability was not correlated with BMI, but pontine SERT availability was found to be positively correlated with BMI. SERT availability was higher in men, but DAT availability was not.

Obesity arises from energy imbalance, whereby energy intake exceeds energy expenditure^21^. This imbalance can be triggered by the internal state of the caloric equation (homeostasis) and by non-homeostatic factors, such as, social, cultural, psychological, environmental factors, food type and the amount consumed^22–24^. In industrialized countries where foods are plentiful, food palatability in increases food intake via a reward mechanism^25^. Dopamine is a neurotransmitter that modulates reward^9^. Repeated exposure to a food reward, reduces the activation of dopamine and induces habituation^26^, as this blunted activation can trigger compensatory overeating^27^. Therefore, decreased sensitivity to the rewarding effects of food consumption due to reduced dopaminergic neuron activation develops in obesity^28^. In this regard, previous studies have focused on the role of DAT and SERT in obesity. Of three studies that investigated the correlation between BMI and DAT^29–31^, one study, in which ^99m^Tc-TRODAT was used, reported a significant correlation coefficient of −0.44^29^. However, in most studies no association was found between BMI and DAT availability^30,31^, which is consistent with our observations. DAT controls extracellular dopamine levels by selectively uptaking dopamine into presynaptic neurons^32^. However, we observed no significant correlation between DAT and BMI. It has been suggested postsynaptically located dopamine receptor might play a dominant role in the reward system^33^. Although previous researches have mainly focused on the role played by dopamine in the reward system, serotonin is also known to play an important role in reward processing^34^. In a previous study, a negative correlation was observed between SERT and BMI was reported using global neocortex, midbrain, and striatum as target regions and the SERT selective radiotracer, ^11^C-DASB^35^. However, a voxel-based analysis of ^123^I-FP-CIT found a positive correlation between BMI and SERT availability in thalamus^36^, and Versteeg RI *et al*.^31^ and Hesses S *et al*.^37^ found no significant association between SERT availability and BMI. In the present study, SERT availability in pons was positively correlated with BMI. Higher SERT recruitment may be a consequence of higher serotonin recruitment due to food overload or overactive reward and homeostatic circuits^38^. Interestingly, SERT availabilities in midbrain were negatively and positively correlated with BMIs in obese and non-obese subjects, respectively. BMI might reflect a different role in predicting serotonergic tonus in midbrain. Thus, we hypothesized that SERT availability increases with BMI in response to serotonin in non-obese subjects. Hinderberger P *et al*.^39^ found SERT availability in the nucleus accumbens was negatively correlated in non-obese subjects and positively correlated in obese subjects with serum brain-derived neurotrophic factor levels, which are negatively correlated with BMI^40^. Therefore, SERT availability in the nucleus accumbens might present similar slopes to those observed in the present study. Serotonin receptor availability is negative correlated with serotonin levels^41^, and SERT availabilities in severely obese subjects were reported to be no different from those of lean subjects^37^, but those of overweight/moderately obese subjects were higher than those of lean subjects^42^. van Galen *et al*. suggested an inversed parabolic relationship between SERT availability and serotonin levels^43^, which is consistent with our findings. However, no previous study has reported a significant link between BMI and SERT availability^42,43^, and thus, this is the first study to describe the effect of obesity on midbrain SERT availability.

Because there is no direct means of measuring dopamine or serotonin concentrations in human brain, we chose to examine DAT and SERT availabilities as determined by ^123^I-FP-CIT SPECT^44^. DAT and SERT availabilities are influenced by radioligand affinity and are sensitive to changes in neurotransmitter concentration, and thus, these factors must be taken into account when interpreting neuroimages^44^. Furthermore, some controversy exists regarding the interpretations of results obtained using radiopharmaceuticals^44^. For example, an increase in SERT can be interpreted as an increase in serotonin level in synapses, or as increased serotonin clearance from extracellular regions in the brain, and thus, interpretations of the opposite effect of obesity on midbrain SERT availability may be less than straightforward.

Age related decreases in DAT^45^ and SERT^45,46^ have been well documented, and were also observed in the present study. However, DAT and SERT availability differences are controversial. Previous studies have reported higher SBRs in striatum^47^ and thalamus^15^ in females, but higher SBRs in pons in males^15^. In the present study, SERT availability was higher in males, but DAT availability was similar in men and women. Therefore, although sex hormones affect serotonin^48^ and dopamine^49^, serotonin neurotransmission may be more susceptible to gender.

This study has several limitations that warrant consideration. First, although this is the largest ^123^I-FP-CIT SPECT study conducted on the topic, data acquisition procedures at multiple sites may have been differed. Second, for normal subjects included in PPMI, ^123^I-FP-CIT SPECT was performed once at baseline enrollment, and thus, further longitudinal studies are needed to investigate the association between the availabilities of DAT and SERT and subject weight changes. Third, we investigated the associations between neuroimaging findings and BMI, and thus, additional studies are needed to investigate the effects of food intake, food stimulation, and glucose loading in obese and lean subjects. Forth we used BMI to define obesity and although BMI is the most commonly used parameter, it might not be **(**is not**?)** directly related to body fat levels^50^.

In conclusion, obesity has an effect on midbrain SERT availability. In addition, BMIs were not found to be correlated with striatal DAT availability, but rather with pontine SERT availability. Furthermore, SERT availability was greater in men, whereas DAT availability was similar in men and women.
